# Remodeling of Neurotransmission, Chemokine, and PI3K-AKT Signaling Genomic Fabrics in Neuropsychiatric Systemic Lupus Erythematosus

**DOI:** 10.3390/genes12020251

**Published:** 2021-02-10

**Authors:** Dumitru Iacobas, Jing Wen, Sanda Iacobas, Noa Schwartz, Chaim Putterman

**Affiliations:** 1 Center for Computational Systems Biology, Personalized Genomics Laboratory, Roy G. Perry College of Engineering, Prairie View A & M University, Prairie View, TX 77446, USA; daiacobas@pvamu.edu; 2 DP Purpura Department of Neuroscience, Albert Einstein College of Medicine, Bronx, NY 10461, USA; 3 Department of Medicine (Rheumatology), Albert Einstein College of Medicine, Bronx, NY 10461, USA; jing.wen1@rutgers.edu (J.W.); nshwartz@montefiore.org (N.S.); 4 Department of Pathology, New York Medical College, Valhalla, NY 10595, USA; sandaiacobas@gmail.com; 5 Department of Microbiology & Immunology, Albert Einstein College of Medicine, Bronx, NY 10461, USA; 6 Azrieli Faculty of Medicine, Bar-Ilan University, Zefat 52100, Israel; 7 Galilee Medical Center, Research Institute, Nahariya 22100, Israel

**Keywords:** neuropsychiatric lupus, TWEAK, Tnfsf12, Fn14, Tnfrsf12a, Akt2, PI3K-AKT pathway, *Adcy3*

## Abstract

Cognitive dysfunction and mood changes are prevalent and especially taxing issues for patients with systemic lupus erythematosus (SLE). Tumor necrosis factor (TNF)-like weak inducer of apoptosis (TWEAK) and its cognate receptor Fn14 have been shown to play an important role in neurocognitive dysfunction in murine lupus. We profiled and compared gene expression in the cortices of MRL/+, MRL/*lpr* (that manifest lupus-like phenotype) and MRL/*lpr*-Fn14 knockout (Fn14ko) adult female mice to determine the transcriptomic impact of TWEAK/Fn14 on cortical gene expression in lupus. We found that the TWEAK/Fn14 pathway strongly affects the expression level, variability and coordination of the genomic fabrics responsible for neurotransmission and chemokine signaling. Dysregulation of the Phosphoinositide 3-kinase (PI3K)-AKT pathway in the MRL/*lpr* lupus strain compared with the MRL/+ control and Fn14ko mice was particularly prominent and, therefore, promising as a potential therapeutic target, although the complexity of the transcriptomic fabric highlights important considerations in in vivo experimental models.

## 1. Introduction

Systemic lupus erythematosus (SLE) is an antibody-driven, autoimmune disease that can potentially affect all organ systems, ranging from skin and joints, to kidneys, heart, and brain. Neuropsychiatric manifestations are among the most prevalent, affecting 20–40% of patients, but continue to be under-recognized and under-addressed [1]. The American College of Rheumatology (ACR) ad-hoc committee identified 19 neuropsychiatric syndromes that occur in SLE, ranging from seizures, chorea, and acute confusional state, to more subtle headaches, mood changes, and cognitive dysfunction [2]. Attributing neuropsychiatric manifestations to SLE, however, is often challenging as they can present in isolation and even predate SLE diagnosis, be indistinguishable in terms of presentation from non-SLE causes, may be associated with medication side effects, or can be attributed to a chronic disease state [3]. Nevertheless, support for neuropsychiatric SLE (NPSLE) manifestations as reflecting primary disease processes come from the fact that they often appear early in the disease course (before neurotoxic or neuro-affective medication administration, or prolonged disease), advanced imaging techniques present similar features among many of the patients, and SLE animal models manifest similar neurocognitive changes. Advancements in the understanding of the etiology of NPSLE began to emerge in the last decade, providing first glimpses as to the inflammatory effects of SLE in the brain, as well as their impact on neurocognition [3]. Furthering our understanding of the underlying processes will enhance our ability to diagnose NPSLE symptoms early, attribute them accurately and promptly, and potentially identify and allow the tailoring of specific treatment regimens.

MRL/MpJ-*Fas^lpr/lpr^* (MRL/*lpr*) lupus prone mice have a loss-of-function mutation in the *Fas* gene, superimposed on a complex MRL background. These mice manifest a lupus-like phenotype, including anti-nuclear antibody formation, immune-complex mediated glomerular disease, and typical skin manifestations [4]. MRL/*lpr* mice develop a range of cognitive and affective symptoms, including memory deficits and depression-like behavior, with prominent neurobehavioral deficits by 16 weeks [5]. This protracted course, in addition to several additional similarities in the clinical course and histologic presentations to human disease, makes this mouse strain a widespread choice for lupus research in general, and NPSLE, in particular.

Tumor necrosis factor (TNF)-like weak inducer of apoptosis, TWEAK (or TNFSF12), is a secreted member of the TNF-ligand superfamily, with pleotropic effects on multiple cell types, including enhancement of the inflammatory milieu, and context-dependent effects on cell survival and apoptosis. TWEAK and its cognate receptor Fn14 (TNFRSF12A) are expressed in astrocytes, microglia, brain microvascular endothelial cells, and neurons [6,7]. TWEAK/Fn14 interaction activates pro-inflammatory cytokine production, and is known to play an important role in NPSLE. We have shown that Fn14ko mice display significantly less depression and cognitive abnormalities than Fn14-sufficient littermates [8], while human NPSLE is associated with high TWEAK levels in the cerebrospinal fluid (CSF) [9].

Utilizing a novel gene expression analysis to determine the impact of the differential cortical transcriptomic fabric between Fn14-sufficient and Fn14-depleted lupus-prone mice would allow for the identification of crucial signaling pathways activated by the TWEAK/Fn14 interaction, emphasize relevant neurotransmission processes, and potentially identify new promising therapeutic targets.

## 2. Materials and Methods

### 2.1. Animals

MRL/*lpr* mice and Fn14ko (backcross generation #8) littermates were bred at Biogen Idec (Cambridge, MA, USA) and transferred to Albert Einstein College of Medicine (AECOM) at 8–10 weeks of age. Control MRL/*MpJ* (MRL/+) mice were purchased from the Jackson Laboratory (Bar Harbor, ME, USA). Housing conditions were controlled, with a temperature of 21–23 °C and a 12:12 h light:dark cycle. All animal study protocols were approved by the institutional animal care and use committee (IACUC) at AECOM (protocol #20170516).

4 MRL/*lpr*, 4 Fn14ko, and 4 MRL/+ mice were used for these studies. All mice were female, and sacrificed at the diestrus phase of their hormonal cycle. At sacrifice, all were within one week of age (about 12 weeks old), and all were sacrificed within a 2-week time period. Following the sacrifice, the cortex was isolated and immediately processed.

### 2.2. Microarrays

We have used our standard protocol [10] for gene expression profiling. Briefly, the cortex of each of the 4 mice from each group (MRL/+, MRL/lpr, and Fn14ko) was minced in ice and the total RNA was extracted with Qiagen RNeasy mini kit (Qiagen, Germantown, MD, USA). RNA quality was checked with Agilent (Santa Clara, CA, USA) RNA 6000 Nano kit in an Agilent 2100 Bioanalyzer. RNA concentrations before and after reverse transcription in the presence of Cy3/Cy5 dUTP were determined with Thermo Fisher Scientific NanoDrop ND 2000 Spectrophotometer (Waltham, MA, USA). Eight hundred and twenty-five nanograms of differently labeled RNAs (Cy3/Cy5) from two biological replicas of the same phenotype were co-hybridized 17 h over night at 65 °C with microarrays of a 4 × 44 k Agilent 60 mer G2519F mouse chip. The microarrays were scanned with an Agilent G2539A dual laser scanner at 5 µm pixel size/20-bit and raw data extracted with Agilent Feature Extraction software vs. 11.1.1.

### 2.3. Filtering and Normalization

All corrupted spots or those with foreground fluorescence less than twice the background in any of the 12 profiled samples were disregarded from the analysis. Data were normalized according to our standard iterative method [10], alternating intra- and inter-array normalization to the expression level of the median gene until the overall maximum error of estimate becomes less than 5%. We chose the median expression for normalization basis because it is not affected by outliers, while average expression is affected. Owing to the non-uniform (from 1 to 13) number of microarray spots redundantly probing the same transcript, the single gene quantifiers were adjusted as described below to get the maximum accuracy.

### 2.4. Single Gene Quantifiers

The 4-biological replicas design of the experiment provides three independent measures for the expression of every single gene in each condition: average expression level (AVE), relative expression variability (REV), and expression correlation (COR) with each other gene [11]. Biological replicas can be considered as the same system subjected to slightly different environmental conditions. AVE, REV, and COR of gene *i* in the phenotype P (=MRL/+, MRL/*lpr*, Fn14ko) were computed as:

(1) $$\begin{array}{l} AVE_{i}^{\left(P\right)}=\frac{1}{R_{i}}\sum_{k=1}^{R_{i}}\mu_{i,k}^{\left(P\right)}=\frac{1}{R_{i}}\sum_{k=1}^{R_{i}}\underset{\mu_{i,k}^{\left(P\right)}}{\underset{︸}{\left(\frac{1}{4}\sum_{\xi =1}^{4}a_{i,k,\xi }^{\left(P\right)}\right)}}\;,\;where:\\ R_{i}\;=\;\mathrm{number}\;\mathrm{of}\;\mathrm{microarray}\;\mathrm{spots}\;\mathrm{probing}\;\mathrm{redundantly}\;\mathrm{gene}\;i\\ a_{i,k,\xi }^{\left(P\right)}=\mathrm{expression}\;\mathrm{level}\;\mathrm{of}\;\mathrm{gene}\;“i”\;\mathrm{probed}\;\mathrm{by}\;\mathrm{spot}\;“k”\;\mathrm{on}\;\mathrm{biological}\;\mathrm{replica}\;“\xi ”\\ \mu_{i,k}^{\left(P\right)}\;=\;\mathrm{average}\;\mathrm{expression}\;\mathrm{level}\;\mathrm{of}\;\mathrm{gene}\;“i”\;\mathrm{probed}\;\mathrm{by}\;\mathrm{spot}\;“k”\;\mathrm{on}\;\mathrm{all}\;\mathrm{biological}\;\mathrm{replicas}\\ REV_{i}^{\left(P\right)}=\underset{\mathrm{correction}\;\mathrm{coefficient}}{\underset{︸}{\frac{1}{2}\left(\sqrt{\frac{r_{i}}{\chi^{2}\left(r_{i};0.975\right)}}+\sqrt{\frac{r_{i}}{\chi^{2}\left(r_{i};0.025\right)}}\right)}}\sqrt{\underset{\mathrm{pooled}\;\mathrm{CV}}{\underset{︸}{\frac{1}{R_{i}}\sum_{k=1}^{R_{i}}{\left(\frac{s_{ik}^{\left(P\right)}}{\mu_{ik}^{\left(P\right)}}\right)}^{2}}}}\times 100\%\\ \chi^{2}=\mathrm{chi}-\mathrm{square}\;\mathrm{score}\;\mathrm{for}\;r_{i}\;\mathrm{degrees}\;\mathrm{of}\;\mathrm{freedom}\;\mathrm{and}\;\alpha \;=\;0.05\\ s_{ik}=\mathrm{standard}\;\mathrm{deviation}\;\mathrm{of}\;\mathrm{the}\;\mathrm{expression}\;\mathrm{level}\;\mathrm{of}\;\mathrm{gene}\;i\;\mathrm{probed}\;\mathrm{by}\;\mathrm{spot}\;k\\ r_{i}=4R_{i}-1=\;\mathrm{number}\;\mathrm{of}\;\mathrm{degrees}\;\mathrm{of}\;\mathrm{freedom}\\ COR_{i,j}^{\left(P\right)}=\frac{\sum_{k=1}^{R_{i}}\left(\left(\frac{1}{4}\sum_{\xi =1}^{4}a_{i,k,\xi }^{\left(P\right)}-\mu_{i,k}^{\left(P\right)}\right)\left(\frac{1}{4}\sum_{\xi =1}^{4}a_{j,k,\xi }^{\left(P\right)}-\mu_{j,k}^{\left(P\right)}\right)\right)}{\sqrt{\sum_{k=1}^{R_{i}}{\left(\frac{1}{4}\sum_{\xi =1}^{4}a_{i,k,\xi }^{\left(P\right)}-\mu_{i,k}^{\left(P\right)}\right)}^{2}\sum_{k=1}^{R_{i}}{\left(\frac{1}{4}\sum_{\xi =1}^{4}a_{j,k,\xi }^{\left(P\right)}-\mu_{j,k}^{\left(P\right)}\right)}^{2}}}\;(\mathrm{Pearson}\;\mathrm{correlation}\;\mathrm{cefficient}) \end{array}$$

Notes:

- AVE of each gene in a particular phenotype is expressed in units equal to the AVE of the median gene in all biological replicas of that phenotype.
- REV combines the coefficients of variation (CV) of the expression levels of all spots probing redundantly the same gene in the mid-interval chi-square estimate of the pooled CV.
- Synergistically-expressed genes change their expression levels in phase across biological replicas, antagonistically expressed do so in antiphase, while with independently expressed genes, change of expression of one gene has no direct consequences on the other. From this perspective, the upstream activator and downstream activated genes of a functional pathway should be synergistically expressed with the central gene. By contrast, the upstream inhibitor and downstream inhibited genes should be antagonistically expressed with the central gene. Independently expressed genes should not be considered as related to a pathway.
- If a gene is probed by a single spot (most cases) then two genes are *p* < 0.05 significantly synergistically expressed if COR > 0.95, significantly antagonistically expressed if COR < −0.95, and independently expressed if −0.025 < COR < 0.025. The absolute cut-off for significant synergism/antagonism changes with more spots redundantly probing the same transcript: |COR| > 0.707 for two spots, …, |COR| > 0.273 for 13 (maximum) number of spots [12].
- COR was determined by the Anaconda distribution of the Python 3 software “CORRELATION”, described in Reference [13].

Coordination analysis was used to reveal “transcriptomic stoichiometry” [10] within the genomic fabrics and of the fabrics’ interplay [14]. The Principle of Transcriptomic Stoichiometry, an extension of Dalton’s Law of Multiple Proportions from chemistry to networked genes, claims that genes are expressed in well-defined proportions to ensure the best efficiency of the functional pathways [14]. As such, genes encoding interacting products in functional pathways should be coordinately expressed, while independent expression of two genes indicates that their encoded products cannot be directly linked in the same pathway.

### 2.5. Gene Hierarchy and Gene Master Regulator of the Phenotype

Gene biomarkers of a disease are usually selected from the genes with most frequently altered sequence or expression level when comparing a large population of sick persons with a demographically matched population of healthy individuals [15,16]. Frequent alteration indicates that their sequence and/or expression level are weakly protected by cellular homeostatic mechanisms as expected for non-essential players. By contrast, genes in which sequence and/or expression level are critical for cell survival/phenotypic expression/integration into multi-cellular structure are strongly protected, and thereby less alterable in terms of their sequence and/or expression level. Moreover, expression of a critical gene regulates major functional pathways through expression coordination with the pathways’ genes. Therefore, in recent papers [11,13,17], we introduced the Gene Commanding Height (GCH) score, a combined measure of expression control and expression coordination with other genes in the phenotype. In this study, GCH was used to establish the hierarchy of genes in terms of their importance in preserving phenotype P:

(2) $$GCH_{i}^{\left(P\right)}\equiv \frac{{\langle REV\rangle }^{\left(P\right)}}{REV_{i}^{\left(P\right)}}\exp\left(\frac{4}{N}{\sum_{j\in ALL,j\neq i}\left(COR_{ij}^{\left(P\right)}\right)}^{2}-1\right)$$

The hierarchy is topped by the Gene Master Regulator (GMR), which is the most influential gene of the phenotype, and, by consequence, the most legitimate target for gene therapy [18]. With GCH one can also evaluate how influential are some other interesting genes, including the proposed NPSLE biomarkers [16,19], for example. GCH scores of all quantified genes in each of the three phenotypes were determined with the Python software “GENE COMMANDING HEIGHT”, described in Reference [13].

### 2.6. Expression Regulation

Instead of referring the absolute fold-change |*x*| to an arbitrary cut-off (1.5× or 2.0×), a gene was considered as significantly regulated in MRL/*lpr* or Fn14ko with respect to MRL/*+* if |x| exceeded the “CUT” of that particular gene in the compared phenotypes. CUT takes into account the combined contributions of the gene expression variabilities in the compared samples, as well as the technical noises of the probing spots in the two microarrays. Moreover, our significant regulation criterion includes the requirement that the p-value of the heteroscedastic *t*-test of the means’ equality is below 0.05.

(3) $$\begin{array}{l} \left|x_{i}^{\left(P\;\mathrm{vs}\;Q\right)}\right|>CUT_{i}^{\left(P\;\mathrm{vs}\;Q\right)}=1+\frac{1}{100}\sqrt{2\left({\left(REV_{i}^{\left(Q\right)}\right)}^{2}+{\left(REV_{i}^{\left(P\right)}\right)}^{2}\right)}\;\wedge \;p_{i}^{\left(P\;\mathrm{vs}\;Q\right)}<0.05\\ where:\;P\;=\;\mathrm{MRL}/\mathrm{lpr},\;\mathrm{Fn}14\mathrm{ko}\;\wedge Q=\;\mathrm{MRL}/+,\;\mathrm{Fn}14\mathrm{ko}\;\wedge Q\neq P\\ x_{i}^{\left(P\;\mathrm{vs}\;Q\right)}\equiv \{\begin{array}{cc} \frac{\mu_{i}^{\left(P\right)}}{\mu_{i}^{\left(Q\right)}} & ,\;\mathrm{if}\;\mu_{i}^{\left(P\right)}\geq \mu_{i}^{\left(Q\right)}\\ -\frac{\mu_{i}^{\left(Q\right)}}{\mu_{i}^{\left(P\right)}} & ,\;\mathrm{if}\;\mu_{i}^{\left(P\right)}<\mu_{i}^{\left(Q\right)} \end{array}\;\mathrm{expression}\;\mathrm{ratio}\;(\mathrm{negative}\;\mathrm{for}\;\mathrm{down}-\mathrm{regulation})\; \end{array}$$

There are three possible measures of expression regulation: (i) “uniform” by assigning the value +1/−1 to each significantly up-/down-regulated gene, (ii) “expression ratio”, and (iii) “weighted individual (gene) regulation (WIR)” [11]. The uniform measure is the most popular owing to its use in the traditional percentage of up-/down-regulated genes. The uniform measure is restricted to only significantly regulated genes with respect to (mostly arbitrarily introduced) cut-offs for fold-change and/or *p*-value. The uniform measure considers all regulated genes as equal contributors to the transcriptome alteration. Expression ratio discriminates genes’ contributions with respect to their fold-change. However, of the three, *WIR* is the most comprehensive measure because it: (i) is applied to all genes, (ii) weighs the gene’s contribution with its normal expression, (iii) considers the absolute net fold-change of the expression level and (iv) takes into account the statistical confidence in the regulation:

(4) $$\begin{array}{l} WIR_{i}^{\left(P\;\mathrm{vs}\;Q\right)}\equiv AVE_{i}^{\left(Q\right)}\underset{\mathrm{regulation}\;\mathrm{sign}}{\underset{︸}{\frac{x_{i}^{\left(P\;\mathrm{vs}\;Q\right)}}{\left|x_{i}^{\left(P\;\mathrm{vs}\;Q\right)}\right|}}}\underset{\mathrm{absolute}\;\mathrm{net}\;\mathrm{fold}-\mathrm{change}}{\underset{︸}{\left(\left|x_{i}^{\left(P\;\mathrm{vs}\;Q\right)}\right|-1\right)}}\underset{\mathrm{confidence}\;\mathrm{of}\;\mathrm{the}\;\mathrm{regulation}}{\underset{︸}{\left(1-p_{i}^{\left(P\;\mathrm{vs}\;Q\right)}\right)}}\\ where:\;P\;=\;\mathrm{MRL}/\mathrm{lpr},\;\mathrm{Fn}14\mathrm{ko}\;\wedge Q=\;\mathrm{MRL}/+,\;\mathrm{Fn}14\mathrm{ko}\;\wedge Q\neq P \end{array}$$

### 2.7. Analysis of the Genomic Fabrics of Functional Pathways

We view the transcriptome as a multi-dimensional mathematical object [11], subjected to a phenotypically specific dynamic set of expression correlations among the genes associated in partially overlapping genomic fabrics [10]. The genomic fabric of a functional pathway was defined as the transcriptome associated with the most stably expressed and interconnected gene network responsible for that pathway [11]. The three independent characteristics of every quantified gene in each phenotype allowed us to characterize the phenotypic differences based on the remodeling of the genomic fabrics in terms of differential expression profile, differential control, and differential inter-coordination.

Kyoto Encyclopedia of Genes and Genomes (KEGG [20]) was used to identify 133 genes involved in the chemokine signaling pathway (Reference [21], hereafter denoted by CHS) and 274 genes of the Phosphoinositide 3-kinase (PI3K)-AKT signaling pathway (Reference [22], AKT). KEGG was also used to select neurotransmission genes: 100 glutamatergic (Reference [23], GLU), 76 GABAergic (Reference [24], GAB), 97 cholinergic (Reference [25], CHO), 117 dopaminergic (Reference [26], DOP) and 83 serotonergic (Reference [27], SER).

Differential expression of all quantified genes (ALL) or of genes from a selected pathway Γ when comparing the three phenotypes among themselves were quantified both as percentages of up- and down-regulated genes and with the Weighted Pathway Regulation (WPR [14]):

(5) $$\begin{array}{l} WPR_{\Gamma }^{\left(P\;\mathrm{vs}\;Q\right)}\equiv \sqrt{\frac{1}{Card(\Gamma )}\sum_{i=1}^{Card(\Gamma )}{\left(WIR_{i\in \Gamma }^{\left(P\;\mathrm{vs}\;Q\right)}\right)}^{2}}\;,\;where:\;\\ P\;=\;\mathrm{MRL}/\mathrm{lpr},\;\mathrm{Fn}14\mathrm{ko}\;\wedge Q=\;\mathrm{MRL}/+,\;\mathrm{Fn}14\mathrm{ko}\;\wedge Q\neq P\\ \Gamma =ALL,CHS,AKT,GLU,GAB,CHO,DOP,SER\\ Card(\Gamma )=\;\mathrm{number}\;\mathrm{of}\;\mathrm{quantified}\;\mathrm{genes}\;\mathrm{in}\;\mathrm{the}\;\mathrm{pathway}\;\Gamma \end{array}$$

## 3. Results

Raw and normalized gene expression data were deposited and are publicly accessible in the Gene Expression Omnibus (GEO) of the National Center for Biotechnology Information (NCBI) (Remodeling of Neurotransmission and Chemokine Signaling Genomic Fabrics in Neuropsychiatric Systemic Lupus Erythematosus. Available online: https://www.ncbi.nlm.nih.gov/geo/query/acc.cgi?acc=GSE164140 (Accessed: 10 January 2021). In total we quantified 16,989 unigenes in all 12 profiled samples, with 16,989 average expression level (AVE), 16,989 relative expression variability (REV), and 144,304,566 expression correlation (COR) values for each phenotype.

### 3.1. Three Independent Characteristics for Every Gene

**Figure 1:**
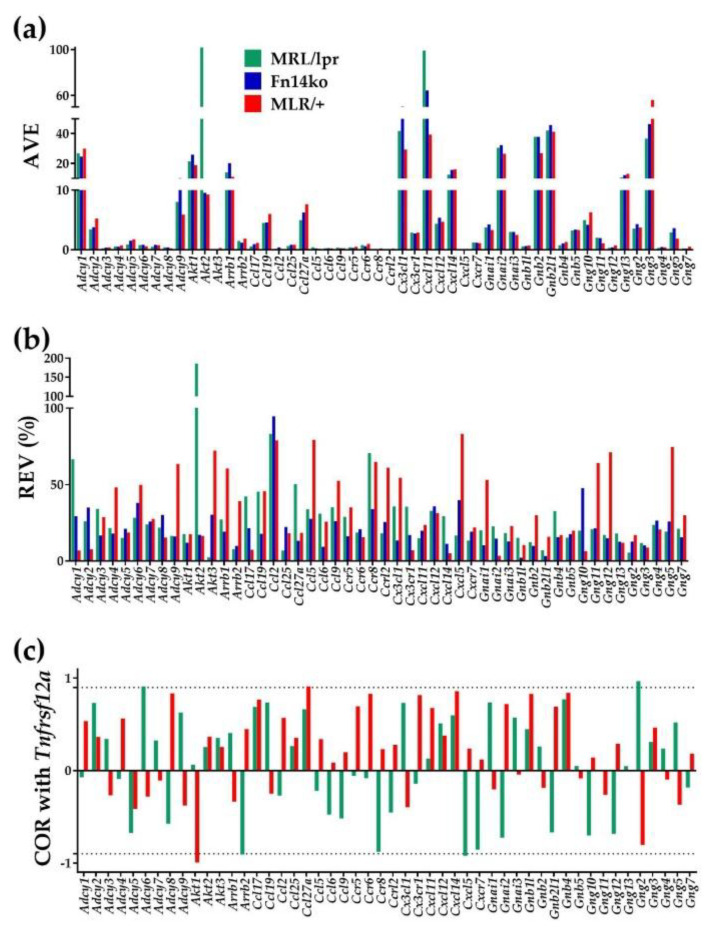
Three independent characteristics of 50 representative genes involved in chemokine signaling (**a**) Average Expression levels (AVE) as multiples of the median average expression levels of all quantified genes. (**b**) Relative Expression Variability (REV). (**c**) Expression correlation (COR) of each gene with *Tnfrsf12a (Fn14)*. Dotted lines indicate the interval outside which the correlations are statistically significant. Notes: (1) AVE is the pool estimate of the expression level of all spots probing the same transcript. (2) REV is the mid-interval chi-square estimate of the coefficient of variation accounting for the non-uniform number of spots redundantly probing the same transcript. (3) Pearson correlation coefficient and its statistical significance were determined for each redundantly probed gene pair in each phenotype by the Python 3 software presented in Reference [13].

Figure 1 presents the three independent characteristics (AVE, REV, and COR) of 50 genes involved in the KEGG-derived chemokine signaling pathway [20]. The independence of the three features was statistically significant, with p-values ranging from 5 × 10^−4^ to 3 × 10^−5^. Figure 1c illustrates the expression correlation of each gene to *Tnfrsf12a* (a.k.a. *Fn14*). Because Fn14ko mice are deficient in *Fn14*, they are not included in this analysis.

The most notable changes appreciated from the AVE and REV analyses in Figure 1 are the gene expression differences of *Akt2* (thymoma viral proto-oncogene 2; a serine/threonine protein kinase that is important in the regulation of glucose uptake, as well as in cell survival, proliferation, growth, and angiogenesis) among the different experimental models. Cortical tissue from MRL/*lpr* mice demonstrated significant upregulation of *Akt2*, as well as highly increased REV, compared with the MRL/+ control. *Akt2* expression and relative variability in Fn14ko is similar to that in the MRL/+ control, indicating that Fn14 deficiency corrects the aberrancy in *Akt2* expression and variability. However, COR analysis surprisingly did not demonstrate a significant correlation between *Fn14* expression and *Akt2*. This highlights the complexity of signaling pathways, and the study of gene expression in general, as expression of a gene of a specific receptor and the activation of this receptor with its downstream effects are separate and sometime independent processes.

### 3.2. Three Measures of Expression Level Regulation of Individual Genes

**Figure 2:**
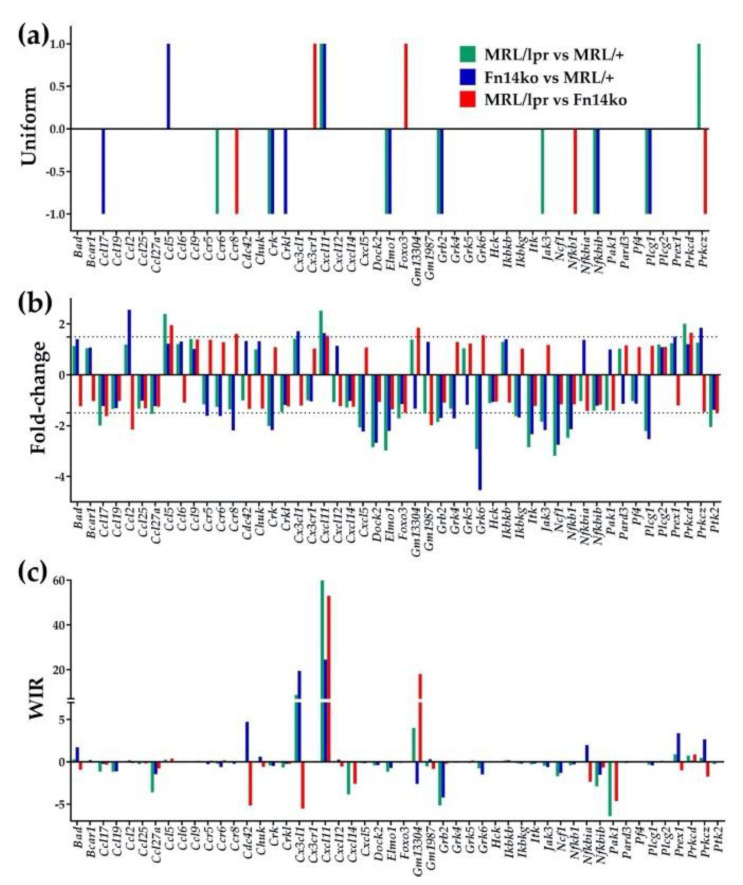
Three measures of comparative expression regulation illustrated in a sample of chemokine-signaling genes. (**a**) Uniform (+1/−1 for up-/down-regulated genes). (**b**) Fold-change (negative for down-regulation). (**c**) Weighted Individual (gene) Regulation (WIR), or the weighted contribution of each gene to the transcriptome. A gene was considered as significantly differentially expressed between two phenotypes if: (1) its absolute fold-change exceeded the cut-off for that gene, considering the contributions of both technical noise and biological variability, and (2) the *p*-value of the heteroscedastic *t*-test of the means’ equality was <0.05.

Figure 2 shows three ways of presenting gene expression level regulation: (a) uniform +1/−1 (upregulation/downregulation) statistically significant contribution to the transcriptomic differences, (b) expression ratio, or “fold-change”, and (c) weighted individual gene regulation (WIR). For this illustration, we chose 50 representative genes involved in the chemokine signaling pathway. As shown in Figure 2a, the “uniform” presentation only indicates whether these genes are significantly upregulated or downregulated but does not provide any further means of differentiating between the genes. At the same time, both the fold-change in expression level (Figure 2b) and WIR (Figure 2c) do. For example, *Cxcl11* (C-X-C motif chemokine 11; an interferon-inducible T-cell chemoattractant) is upregulated in all three comparisons, but it is through the fold-change analysis that we can understand the magnitude of the difference in expression levels (2.52× in MRL/*lpr* vs. MRL/+; 1.64× in Fn14ko vs. MRL/+; and 1.52× in MRL/*lpr* vs. Fn14ko). WIR analysis further demonstrates that, within this selection of genes, *Cxcl11* has the largest positive contribution to the MRL/*lpr* transcriptome (59.87), and somewhat less so to the Fn14ko (24.55) compared to background controls.

### 3.3. Regulation of Signaling Pathways and NPSLE Biomarkers

**Figure 3:**
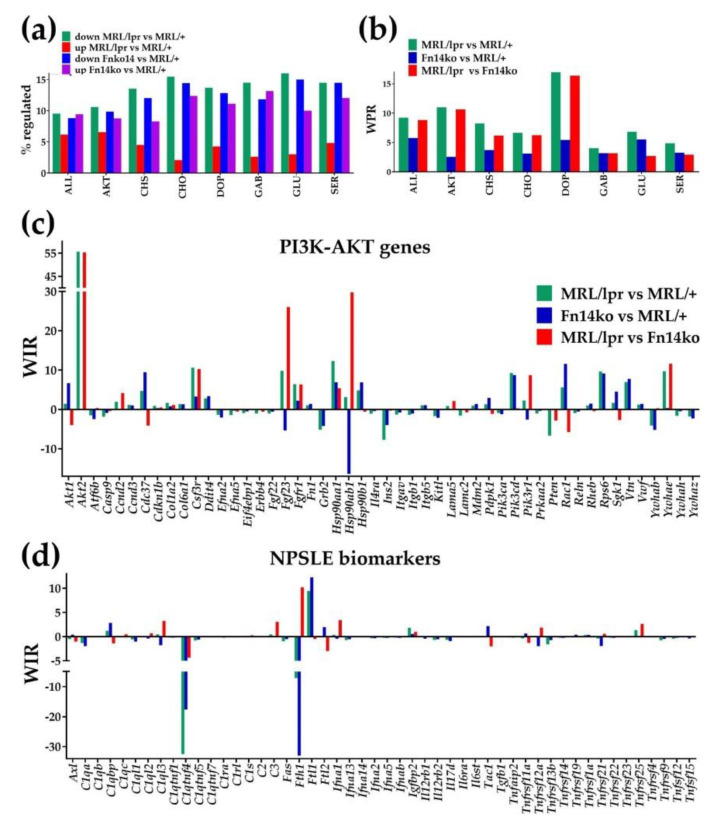
Transcriptomic regulation of selected signaling pathways, PI3K-AKT individual genes, and neuropsychiatric systemic lupus erythematosus (NPSLE) biomarkers. (**a**) Percentages of up- and down-regulated genes. ALL = all genes, AKT = PI3K-AKT signaling pathway, CHS = chemokine signaling pathway, CHO = cholinergic transmission, DOP = dopaminergic transmission, GAB = GABAergic transmission, GLU = glutamatergic transmission, SER = serotonergic transmission. (**b**) Weighted Pathway Regulation (WPR) of individual pathways and the WPR ratio of the two profiled mice models. (**c**) Weighted Individual (gene) Regulation (WIR) of selected genes involved in the PI3K-AKT signaling pathway but not in neurotransmission (excepting *Akt2*). (**d**) WIR scores of NPSLE biomarkers in all three comparisons among the phenotypes.

Going beyond individual genes, we proceeded to examine relevant pathways that may be differentially regulated in the setting of NPSLE. Increased levels of pro-inflammatory cytokines and chemokines in the brains of SLE patients are thought to play an important role in the development of neuropsychiatric manifestations [3]. In addition, neurotransmission is often the final outcome of signaling pathways in the brain and a key shaper of neuronal networks, which have been shown to be significantly altered in the context of NPSLE [28]. We therefore chose to focus on major KEGG-derived neurotransmission and chemokine signaling pathways that may potentially spotlight central pathogenic mechanisms of NPSLE. The apparent importance of *Akt2* in the MRL/*lpr* model, as shown in Figure 1 and Figure 3b, also directed us to examine the role of the PI3K-AKT pathway.

**Figure 4:**
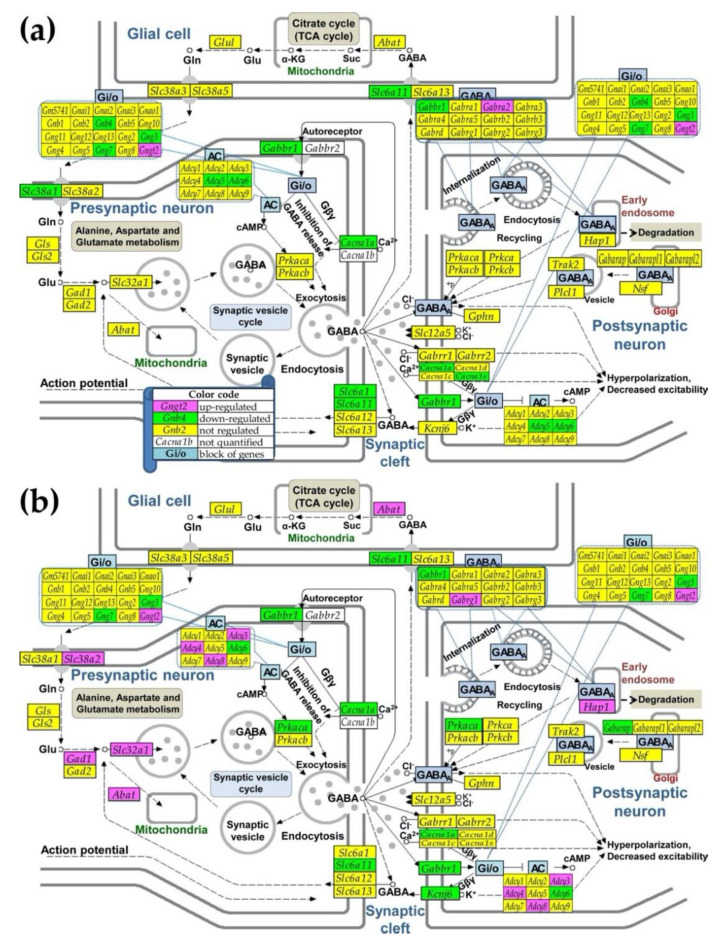
Regulation of the KEGG-determined GABAergic transmission in (**a**) Fn14ko and (**b**) MRL/*lpr* with respect to MRL/*+*. Owing to space constraints, some genes encoding proteins with similar functions were grouped in blocks denoted by KEGG as AC (adenylate cyclase), Gi/o (guanine nucleotide binding proteins), and GABA_A_ (γ-aminobutyric acid receptors). Note the non-uniform (even opposite in some cases) regulation of genes from the same block.

Table 1 lists significantly regulated neurotransmission genes in the MRL/*lpr* mice cortex and their corresponding expression ratios and WIRs in the Fn14ko mice, both with respect to the MRL/+ control. Of specific interest are the significantly down-regulated or up-regulated genes in MRL/*lpr*, yet without a similar effect in the Fn14ko mice, as these genes are likely more directly related to TWEAK/Fn14 activity. *Adcy3* (adenylate cyclase 3; catalyzes the formation of the signaling molecule cyclic adenosine monophosphate (cAMP); WIR = 165.74); and *Akt2* (WIR = 55.62) were most notable in their level of contribution to the transcriptome of MRL/*lpr* compared to MRL/+. No prior association has been made between these 2 genes, but it is notable that both encode for enzymes that are involved in glucose metabolism [29]. Interestingly, while neither gene has been studied in the setting of NPSLE, insulin resistance has been implicated in neurocognitive dysfunction [30]. Of note, two other main contributors to the lupus phenotype are also part of the PI3K-AKT pathway: *Gm2436* (WIR = 19.88) and *Gng3* (WIR = −30.02). As an illustration of the complex interplay of the regulated genes within a neurotransmission pathway, we present a schematic of the GABAergic pathway (Figure 4).

Figure 3a shows the percentages of up- and down-regulated genes of each pathway compared with MRL/+, and Figure 3b displays the weighted pathway regulation (WPR) differences among the 3 phenotypes for all genes (ALL), as well as for the investigated neurotransmission, chemokine, and PI3K-AKT signaling pathways. In general, compared to the MRL/+ control, fewer genes were upregulated in the MRL/*lpr* model than in Fn14ko, without notable differences in the downregulated genes. At the same time, based on the WPR (Figure 3b), the differences between the two controls (Fn14ko and MRL/+) are substantially smaller than those between the lupus-prone mouse (MRL/lpr) and the two controls. The dopaminergic pathway stands out as the most affected neurotransmission pathway in the MRL/*lpr* phenotype, although others show similar trend, as demonstrated in Table 1 and Figure 3b.

WIR analysis of genes from the PI3K-AKT (AKT) pathway that are not part of neurotransmission pathways is shown in Figure 3c. Most impressively, WIR of *Akt2* in the MRL/*lpr* model is considerably high with respect to both MRL/+ and Fn14ko, while it is practically null (0.06) in the Fn14ko mice compared to MRL/+. These results indicate the importance of *Akt2* in regulating the MRL/*lpr* lupus-prone phenotype, and highlight the role of TWEAK/Fn14 activation.

Figure 3d presents the WIRs of genes previously identified as possible NPSLE biomarkers [16] (extensive review of potential biomarkers can be found in Reference [30]). *C1qtnf4* (C1q and tumor necrosis factor related protein 4; known to have enriched expression in the brain, and plays a role in the regulation of inflammation) had the largest (negative) contribution to the transcriptomic alterations in the cortex of MRL/*lpr* with respect to MRL/+ control. However, in general, most biomarker genes do not seem to make a significant contribution to the lupus-prone transcriptome of the MRL/*lpr* mouse.

### 3.4. Gene Hierarchy

**Figure 5:**
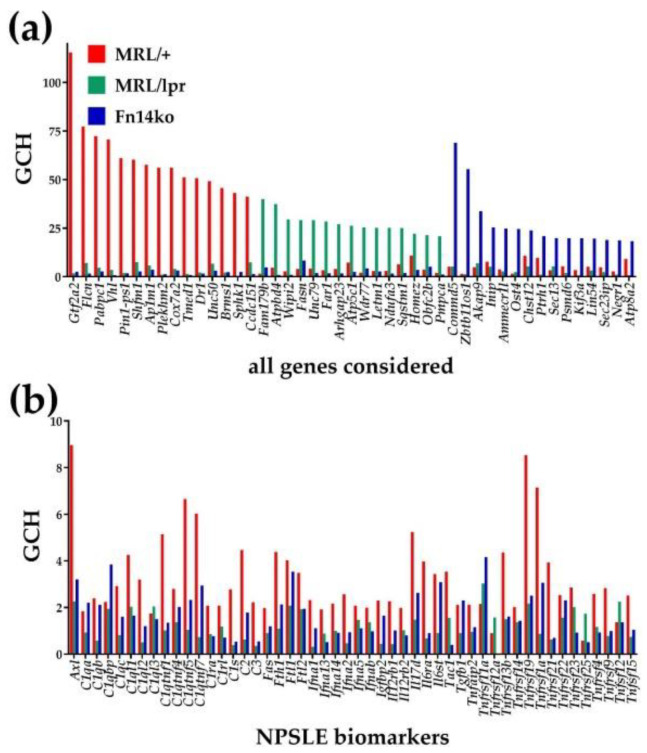
Gene Commanding Height (GCH) scores of: (**a**) top 15 genes in the cortex of each mouse model, (**b**) 50 potential NPSLE biomarkers.

As described previously [13], genes that are important to the normal functioning of the system are generally more preserved, both in term of expression level and sequence. In addition, the more important the gene, the more related genes are dependent on it. The gene commanding height (GCH) is a combined measure of expression control and coordination with many other genes, that can be useful to predict and identify the crucial genes involved in a particular system, or the Gene Master Regulators (GMRs). Figure 5a presents the top 15 genes in each strain’s cortex, and their GCH scores in the other strains. Of main interest are the GMRs in the MRL/+ phenotype, that are likely important in preserving the mice health, and are less regulated or protected in the MRL/*lpr* lupus-prone model. For example, the top GMR identified in the MRL/+ mice is *Gtf2a2* (general transcription factor IIA subunit 2), which has been previously implicated as potential gene regulator in the context of human SLE [31]. For comparison, we present in Figure 5b the GCH scores of the previously introduced potential biomarkers for NPSLE. It is evident that none of these putative biomarkers has a significant GCH (note the difference in magnitude compared to the GCH scores of the genes in Figure 5a), rendering them unlikely to be major lupus susceptibility genes, or serve as targets for effective therapy.

### 3.5. Remodeling of the Transcriptomic Networks

**Figure 6:**
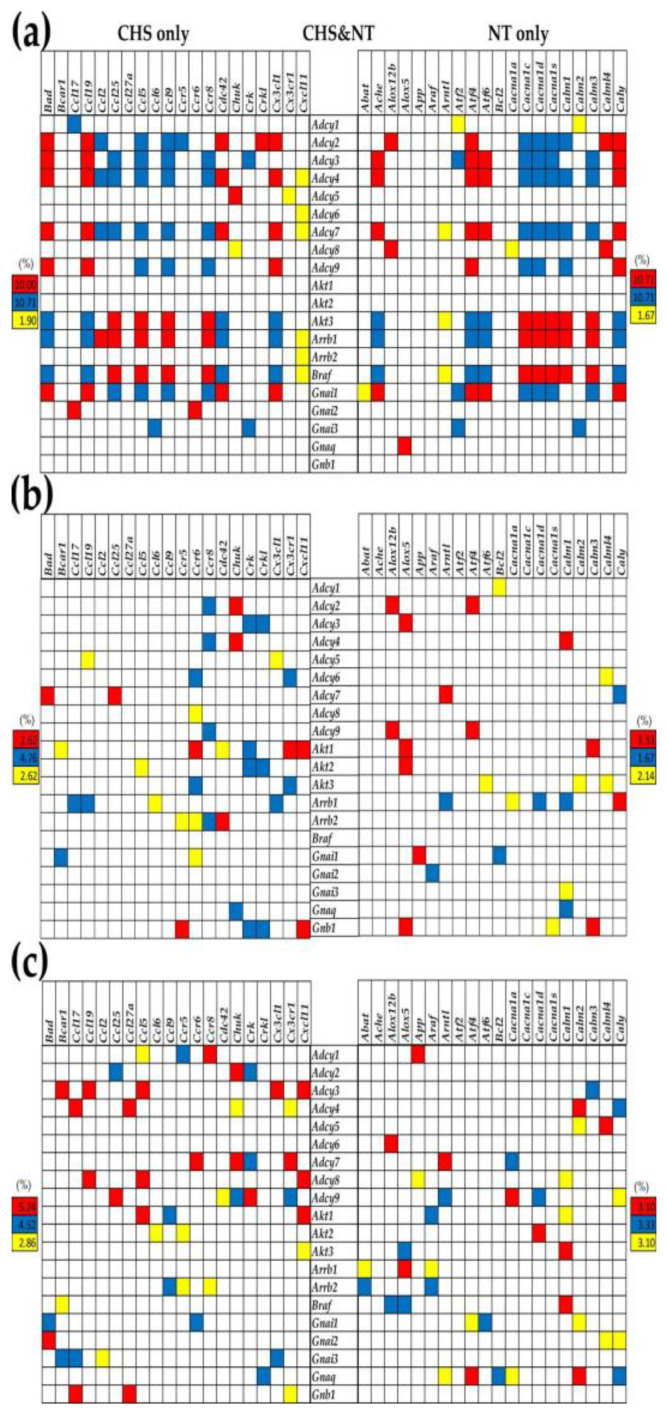
Transcriptomic network connecting chemokine signaling with neurotransmission genes via common genes of the two pathways in (**a**) MRL/*+*, (**b**) MRL/*lpr*, (**c**) Fn14ko brains. A red/blue/yellow square indicates statistically significant synergistic/antagonistic/independent expression of the genes labeling the crossing row and column. A blank square indicates not significant correlation between the expressions of the two genes.

Genes are part of a complex, multi-dimensional system that involves intricate connections among its parts. The transcriptome, therefore, should be examined as a dynamic network, rather than a compilation of independent genes. Figure 6 presents the statistically significant (*p* < 0.05) transcriptomic network of each mouse model, meshing chemokine signaling genes (CHS) with neurotransmission genes (NT), as mediated by genes that are related to both (CHS and NT). Of note is the substantially different transcriptomic organization in the MRL/+ control (Figure 6a) compared with the MRL/*lpr* (Figure 6b) and Fn14ko (Figure 6c), indicating extensive coordination between the CHS and NT pathways in the controls. At the same time, remodeling of the transcriptomic network in Fn14ko compared to MRL/*lpr* mice, illustrates the fact that single-gene knockout models are not quite “single-gene altered systems”, as the coordination of many genes are potentially affected.

**Figure 7:**
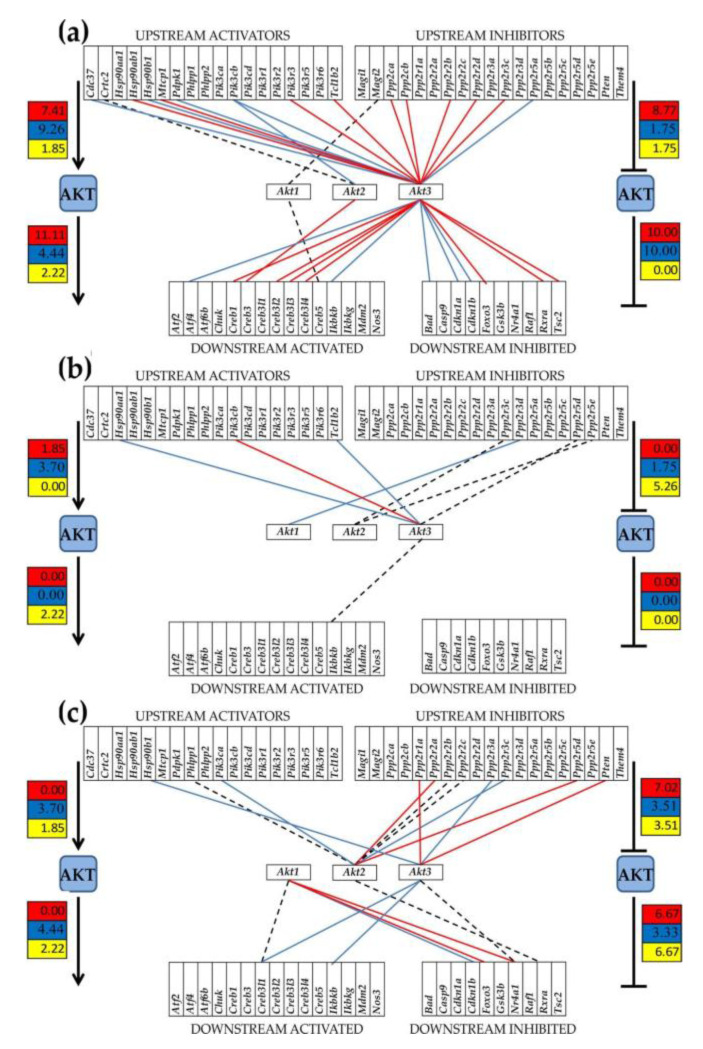
Statistically significant expression correlation of the AKT block of genes (*Akt1, Akt2, Akt3*) with KEGG software identified upstream and downstream genes in (**a**) MRL/+, (**b**) MRL/*lpr*, (**c**) Fn14ko mouse cortices. Red/blue/black lines indicate synergistic/antagonistic/independent expression of the linked genes. Missing gene interlinks mean that the expression correlation was not statistically significant. Numbers on the icons indicate percentage of: statistically significant (*p* < 0.05) synergistically (red background), antagonistically (blue background), or independently (yellow background) expressed gene pairs in the corresponding group of AKT partners.

*Akt2* is a central component of the PI3K-AKT intracellular pathway, which can be regulated by TWEAK/Fn14 in certain clinical situations [32]. As our data suggest, *Akt2* in the MRL/*lpr* mice is significantly overexpressed, compared with both Fn14ko and background control. To get an idea of the general expression and activity of the PI3K-AKT pathway as a whole in the cortex of the studied mice, we examined the predicted gene activation and inhibition based on KEGG-determined PI3K-AKT functional pathway data [20] and assessed the gene expression coordination in each of the three models (Figure 7). For this, we have selected all upstream genes considered by KEGG as activators or inhibitors of the AKT block (*Akt1*, *Akt2*, *Akt3*) and all downstream genes that are activated or inhibited by the AKT block. If the KEGG-determined PI3K-AKT pathway is accurate, then most activator and activated genes should be synergistically expressed with the three Akt genes and most inhibitor and inhibited genes should be antagonistically expressed with the Akt genes. While coordination of some upstream and downstream genes may not be statistically significant, no activator/activated gene should be significantly antagonistically expressed, and no inhibitor/inhibited gene should be synergistically expressed with any of the AKT genes. Interestingly, while the KEGG software predicts a universal pathway for all mouse phenotypes, we found that the three phenotypes have substantially different total numbers and distributions of the gene expression correlations: MRL/+ (17 synergistic, 11 antagonistic, 3 independent), MRL/*lpr* (1 synergistic, 3 antagonistic, 4 independent), and Fn14ko (6 synergistic, 7 antagonistic, 6 independent). Moreover, some of the predicted positive correlations were reversed, including that of the activator *Hsp90b1* and activated *Ikbk* in both MRL/+ and Fn14koMRL. Other gene pairs, such as *Akt3-Tcl1b2* (T-cell leukemia/lymphoma 1B, 2), confirms the KEGG (positive) prediction in one phenotype (MRL/+) but shows the opposite correlation in another phenotype (MRL/*lpr*). In general, the MRL/+ controls displayed closer pathway correlations with that predicted by the KEGG software (Figure 7a), while the MRL/*lpr* mice displayed gross discrepancies (Figure 7b). As shown in Figure 7c, the Fn14ko mice had some recovery of the expected pathway associations but not fully to the control levels. This phenotype dependency of the functional pathways corroborates our previous observations in rat lungs [18], connexin deficient mouse brain [33], heart [34], astrocytes [35], and mouse spinal cord [36], among others.

## 4. Discussion

In this study, we utilized a novel method of RNA expression analysis, whereby gene regulation is evaluated in a multi-dimensional context. Each gene is evaluated not only by its own up-/or down-regulation, but by way of its impact on other genes within the system. As genes do not function in a vacuum, this method is thought to identify the more significant changes that are likely to be critical to the studied processes. Here, we present an analysis of genetic changes in the cortex of NPSLE-prone mice, with an emphasis on the role of TWEAK/Fn14 signaling. We examined the well characterized MRL/*lpr* strain, as compared to Fn14-deficient MRL/*lpr* mice and the healthy MRL/+ background control. Although gene expression profiles are descriptive in nature, the analysis of their correlations may support mechanistic explanations of pathophysiological observations and suggest further routes of investigation.

TWEAK/Fn14 interactions have been shown to play an important role in SLE, including kidney and skin disease [37,38], as well as neuropsychiatric manifestations [7]. We have previously demonstrated that Fn14-deficient MRL/*lpr* mice display significantly less depression and cognitive decline than Fn14-sufficient MRL/*lpr* mice [8]. Moreover, human NPSLE is associated with increased TWEAK levels in the CSF [9]. Nevertheless, the direct pathogenic effect of TWEAK/Fn14 on the NPSLE brain has yet to be fully characterized. In designing this study, we aimed to describe the differential transcriptomic profile and organization between the lupus-prone MRL/*lpr*, Fn14ko, and the MRL/+ background control. In addition, we tried to focus on the signaling pathways by which TWEAK/Fn14 activation operates to elucidate its pathogenic effects.

We identified *Akt2* as a gene that is highly overexpressed in the brain of MRL/*lpr* mice. According to WIR analysis, *Akt2* is the major contributor to the alteration of the PI3K-AKT signaling pathway, that is central to the model’s phenotype. The PI3K-AKT signaling pathway plays a role in cell growth and survival in health, as well as in a myriad of cancers, when dysregulated [39,40]. TWEAK/Fn14 activates *Akt* in several organs and disease models, such as in the heart [41] and skeletal muscles [42], as well as in several tumors, including gliomas [32,43,44]. Nevertheless, our study provides the first evidence of its role in NPSLE. Pathway analysis places *Akt2* in both the dopaminergic and cholinergic neurotransmitter pathways, indicating its importance in brain homeostasis. As *Akt2* is important in cell survival and tissue repair, activation of this pathway may be a compensatory mechanism of the NPSLE brain to repair local damage; in contrast, it is possible that aberrant *Akt2* expression in and of itself is pathogenic to brain tissue and/or function through its direct effects on neurotransmission. Based on our results at this time it is only possible to deduce an association between the *Akt2* gene and TWEAK/Fn14; additional studies will be required into the PI3K-AKT signaling pathway and its role in the context of NPSLE to directly determine causality.

It is important to point out that, while *Akt2* has been shown to be significantly upregulated, as well as a focus of increased variability in the MRL/*lpr* brain compared with its relative conservation in the Fn14ko and MRL/+ controls (indicating the aberrant nature of its upregulation), the expression of *Akt2* does not seem to be directly correlated with Fn14 expression. This can be due to the difference between expression and activation. While Fn14 may be expressed in similar levels in the brains of MRL/*lpr* and MRL/+ mice, its activation is amplified in the MRL/*lpr* mice due to increased TWEAK levels, thereby affecting the downstream expression of *Akt2*. Previous findings in other pathological scenarios of the association between TWEAK/Fn14 and the PI3K-AKT pathway [41,42,43,44,45] support the likelihood of this hypothesis.

Another gene that was highly expressed and showed significant impact on the transcriptomic modifications of the MRL/*lpr* mouse, compared to both the Fn14ko and background control was *Adcy3*. *Adcy3* localizes to primary cilia throughout the brain [46], and low level of its transcripts are associated with depression [47]. Loss-of-function mutations have been associated with insulin resistance and obesity [29,48,49,50], both shown to directly correlate with cognitive dysfunction [51,52]. In addition, *Adcy3* overexpression has been shown to play a role in cell migration, proliferation, and tumor invasion [53]. While expression of this gene appears to affect neurocognitive function, no association has been previously shown with TWEAK/Fn14, nor within the context of chronic inflammation. Of note, it is also possible that the observed overexpression of this neuroprotective gene in NPSLE-setting may be due to brain compensatory mechanisms, as a response to ongoing local neurotoxic inflammation.

Most of the genes encoding proposed NPSLE biomarkers [16] did not prove to have a significant contribution to the lupus-prone cortical transcriptome of the MRL/*lpr*, compared with its background control. However, an interesting observation regarding *C1qtnf4* may prove useful for future investigations. *C1qtnf4*, an inflammation-regulatory gene that is enriched in the brain tissue, was found to have the largest (inverse) contribution to the cortex transcriptomic alterations in the MRL/*lpr* mouse compared with MRL/+ among all of the studied biomarkers. A large contribution, although not quite similar in magnitude, was demonstrated in the Fn14ko mouse, as well. *C1qtnf4*, therefore, is likely a contributor to the MRL/*lpr* phenotype independent of TWEAK/Fn14 activity. Interestingly, there are reports of a gain-of-function mutation in the *C1qtnf4* gene that correlates with early-onset, severe SLE in human patients [54,55], further supporting a role for the *C1qtnf4* gene in autoimmune homeostasis and aberrant activity in SLE, as suggested by our findings.

In this study we also show significant changes in regulatory genes of multiple neurotransmitter pathways in the brains of the lupus-prone mice, providing evidence for profound changes in brain functioning in NPSLE. This is interesting in light of the fact that MRL/*lpr* mice mainly display NPSLE symptoms, such as depressed mood and neurocognitive decline. Interestingly, these manifestations clinically are those that often prove to be most difficult to attribute to SLE, as they can be subtle and/or associated with other non-inflammatory causes, such as metabolic changes, medication side effects, or functional changes, in response to chronic illness. There have been studies demonstrating changes in SLE patients’ neural networks in association with these particular NPSLE symptoms [28], and our findings provide additional support to substantial changes to the NPSLE brain’s microenvironment.

Finally, in this paper, we present a novel analysis of transcriptomic networks of genes in the brains of our studied mice models. Notably, the transcriptomic network of the Fn14ko mice is appreciably different than those of the MRL/*lpr* model. This indicates that knocking out a single gene in a model can potentially affect a whole network of related genes, something that needs to be considered when comparing the knock-out strain with its background control.

The major limitation of our study comes from the high cellular heterogeneity of the cortex. It is a fact that different cellular phenotypes have different transcriptomic topologies and respond differently to disease. Nonetheless, even cells of the same phenotype are not identical owing to the non-uniform action of epigenetic factors. However, spreading the cortex into monocellular cultures and studying them separately is not the solution because the cellular environment is a major transcriptomic regulator as we have previously shown by profiling the mouse astrocytes and oligodendrocytes when cultured alone and co-cultured in insert systems [10,19]. In addition to the limitation of heterogeneity of cells, we should also point out that mouse models, and particularly the MRL/*lpr* and MRL/+ strains, are often heterogeneous even within their phenotype, with a range of disease activity and extent of organ involvement. Due to the complex nature of this study, we only used 4 mice from each phenotype. To mitigate at least some of the heterogeneity issue, we made sure to focus only on data that had clear, robust differences between the models, beyond just a statistical significance. Another limitation is that it has been demonstrated that not just sex differences but also where the female mice are in their estrous cycles, can affect gene expression in different tissues, such as the heart [56,57]. While we only utilized female mice for this study, and attempted to sacrifice all in the same stage of their estrous cycle, there is always inherent variability in hormonal levels and cycle length between mice, thereby potentially adding a hormone-dependent component to the observed gene expression in our experimental model. Moreover, it would be interesting to see whether the brain gene expression network of male NPSLE mice would differ significantly from that displayed by female mice. Finally, as mentioned previously, this observational study can only describe associations between mice models, gene expression, and signaling pathways, but it cannot prove causality. In future studies, we plan to further confirm these results with real-time PCR of the specific gene transcripts and assessment of local protein levels, while aiming to further elucidate the interplay between TWEAK/Fn14 activation, PI3K-AKT signaling pathway, and the brain microenvironment in NPSLE pathogenesis. Despite the above limitations, the strong evidence provided for new and intriguing pathogenic pathways in NPSLE can make a significant impact on our understanding of the disease, with direct potential for innovative therapeutic targets.

## 5. Conclusions

The localized brain processes underlying neuropsychiatric SLE remain largely unexplained. Our goal in this study was to identify chemokine signaling and neurotransmitter pathways that contribute to NPSLE manifestations. The study focused on TWEAK/Fn14-regulated mechanisms, as this ligand/receptor pair has been shown to play an important role in NPSLE. We utilized a novel high-throughput RNA expression analysis that takes into account the impact of the gene expression level, variability, and inter-coordination within the context of the transcriptome, thereby focusing the attention to critical genes. Our findings suggest a role for TWEAK/Fn14-induced cortical activation of the PI3K-AKT pathway, in addition to identifying multiple neurotransmitter regulatory genes that are dysregulated in the lupus-prone mice. Importantly, we have also shown that the brain transcriptome is highly complex and easily affected by even small changes to specific genes, making in vivo studies challenging to interpret. Our observations need to be followed up by directed investigations into the specific pathways but also serve as an important roadmap for future NPSLE studies.

## Figures and Tables

**Table 1:** Regulation of the Kyoto Encyclopedia of Genes and Genomes (KEGG)-determined neurotransmission genes in MRL/*lpr* and Fn14ko compared with background controls. GLU, glutamatergic; GAB, GABAergic; CHO, cholinergic; DOP, dopaminergic; SER, serotonergic; x, expression ratio; WIR, weighted individual (gene) regulation. Blue background of gene symbols indicates regulated neurotransmission genes that are also involved in the PI3K-AKT signaling pathway, while grey background indicates down-regulation (negative fold-change and WIR) in the indicated comparisons.

Gene	Synapse	Description	MRL/lpr		Fn14ko	
			×	WIR	×	WIR
*Adcy3*	CHO,GAB,GLU	adenylate cyclase 3	18.95	165.74	1.22	3.22
*Adcy5*	CHO,DOP,GAB,GLU,SER	adenylate cyclase 5	−2.00	−1.75		
*Adcy6*	CHO,GAB,GLU	adenylate cyclase 6	−1.89	−19.08	−1.67	−14.38
*Akt2*	CHO,DOP	thymoma viral proto-oncogene 2	11.01	55.62		
*Aft4*	CHO,DOP	activating transcription factor 4			2.52	11.21
*Braf*	SER	Braf transforming gene	−3.47	−0.83	−4.46	−1.18
*Cacna1a*	CHO,DOP,GAB,GLU,SER	calcium channel, voltage-dependent, P/Q type, alpha 1A subunit	−1.84	−1.37	−3.72	−4.48
*Cacna1s*	CHO,GAB,SER	calcium channel, voltage-dependent, L type, alpha 1S subunit	−2.48	−0.54		
*Calm14*	DOP	calmodulin-like 4	1.62	29.72		
*Camk2d*	CHO,DOP	calcium/calmodulin-dependent protein kinase II, delta	−2.24	−0.92		
*Chrm1*	CHO	cholinergic receptor, muscarinic 1, CNS	−1.60	−1.22	−1.53	−1.08
*Comt*	DOP	catechol-O-methyltransferase	−1.83	−1.75		
*Creb3*	CHO,DOP	cAMP responsive element binding protein 3	−1.79	−1.77		
*Cyp2d11*	SER	cytochrome P450, family 2, subfamily d, polypeptide 11	−1.62	−2.52	−1.68	−2.79
*Cyp2d12*	SER	cytochrome P450, family 2, subfamily d, polypeptide 12	−1.61	−0.27	−1.70	−0.32
*Fyn*	CHO	Fyn proto-oncogene	−1.38	−0.85	−1.37	−0.83
*Gabbr1*	GAB	gamma-aminobutyric acid (GABA) B receptor, 1	−1.74	−2.63	−2.16	−4.17
*Gabra2*	GAB	gamma-aminobutyric acid type A receptor subunit alpha 2	1.56	1.25		
*Gabrg1*	GAB	gamma-aminobutyric acid type A receptor subunit gamma 1			2.07	0.73
*Gm2436*	DOP	Predicted gene 2436	1.57	19.88		
*Gnal*	DOP	guanine nucleotide binding protein, alpha stimulating, olfactory type	2.14	7.00	1.73	4.41
*Gng3*	CHO,DOP,GAB,GLU,SER	guanine nucleotide binding protein (G protein), gamma 3	−1.53	−30.02	−1.21	−11.35
*Gng7*	CHO,DOP,GAB,GLU,SER	guanine nucleotide binding protein (G protein), gamma 7	−2.07	−0.56	−2.53	−0.81
*Gngt2*	CHO,DOP,GAB,GLU,SER	guanine nucleotide binding protein (G protein), gamma transducing activity polypeptide 2	1.49	0.20		
*Gria2*	DOP,GLU	glutamate receptor, ionotropic, AMPA2 (alpha 2)	−1.52	−2.69		
*Grin2b*	DOP,GLU	glutamate receptor, ionotropic, NMDA2B (epsilon 2)	1.56	0.15		
*Grm2*	GLU	glutamate receptor, metabotropic 2	−1.87	−3.10	−2.28	−4.56
*Grm4*	GLU	glutamate receptor, metabotropic 4	−3.08	−2.98	−2.90	−2.72
*Homer1*	GLU	homer homolog 1 (Drosophila)	−1.66	−0.63		
*Kcnd2*	SER	potassium voltage-gated channel, Shal-related family, member 2	1.30	1.32		
*Kras*	CHO,SER	v-Ki-ras2 Kirsten rat sarcoma viral oncogene homolog	−1.62	−0.27	−1.51	−0.23
*Mapk11*	DOP	mitogen-activated protein kinase 11	−2.37	−0.45	−2.50	−0.50
*Mapk12*	DOP	mitogen-activated protein kinase 12	−2.96	−3.22	−1.92	−1.49
*Pik3ca*	CHO	phosphatidylinositol 3-kinase, catalytic, alpha polypeptide	−1.95	−0.89	−2.32	−1.23
*Pik3cb*	CHO	phosphatidylinositol 3-kinase, catalytic, beta polypeptide	−1.15	−0.25	−1.75	−1.26
*Plcb3*	CHO,DOP,GLU,SER	phospholipase C, beta 3	1.25	0.28	1.42	0.45
*Plcb4*	CHO,DOP,GLU,SER	phospholipase C, beta 4	−2.34	−1.21	−1.68	−0.63
*Ppp1cb*	DOP	protein phosphatase 1, catalytic subunit, beta isoform	−2.13	−1.69		
*Ppp1cc*	DOP	protein phosphatase 1, catalytic subunit, gamma isoform	−2.02	−0.88	−1.39	−0.33
*Ppp2r2a*	DOP	protein phosphatase 2 (formerly 2A), regulatory subunit B	−1.89	−0.57	−1.88	−0.56
*Ppp2r2c*	DOP	protein phosphatase 2 (formerly 2A), regulatory subunit B (PR 52), gamma isoform	−1.85	−0.64	−1.44	−0.32
*Ppp2r5e*	DOP	protein phosphatase 2, regulatory subunit B (B56), epsilon isoform	−1.80	−0.42	−1.60	−0.32
*Rapgef3*	SER	Rap guanine nucleotide exchange factor (GEF) 3	1.37	0.94	1.68	1.77
*Shank1*	GLU	Rap guanine nucleotide exchange factor (GEF) 3	−2.96	−0.88	−2.32	−0.58
*Slc17a7*	GLU	solute carrier family 17 (sodium-dependent inorganic phosphate cotransporter), member 7	−2.43	−44.12	−2.19	−36.79
*Slc17a8*	GLU	solute carrier family 17 (sodium-dependent inorganic phosphate cotransporter), member 8	−1.42	−0.24	−1.57	−0.33
*Slc38a1*	GAB,GLU	solute carrier family 38, member 1	−1.32	−3.57		
*Slc6a1*	GAB	solute carrier family 6 (neurotransmitter transporter, GABA), member 1	−2.35	−0.76		
*Slc6a11*	GAB	solute carrier family 6 (neurotransmitter transporter, GABA), member 11	−2.55	−6.66	−2.72	−7.35
*Th*	DOP	tyrosine hydroxylase	−2.86	−0.67	−2.40	−0.50

## Data Availability

The data presented in this study are openly accessible in the Gene Expression Omnibus (GEO) of the National Center for Biotechnology Information (NCBI) (Remodeling of Neurotransmission and Chemokine Signaling Genomic Fabrics in Neuropsychiatric Systemic Lupus Erythematosus. Available online: https://www.ncbi.nlm.nih.gov/geo/query/acc.cgi?acc=GSE164140 accessed: 10 January 2021).

## References

[1] Kello N., Anderson E., Diamond B. (2019). Cognitive dysfunction in systemic lupus erythematosus: A case for initiating trials. Arthritis Rheumatol..

[2] Liang M.H., Corzillius M., Bae S., Lew R.A., Fortin P.R., Gordon C., Isenberg D., Alarcon G., Straaton K.V., Denburg J. (1999). The American College of Rheumatology nomenclature and case definitions for neuropsychiatric lupus syndromes. Arthritis Rheumatol..

[3] Schwartz N., Stock A.D., Putterman C. (2019). Neuropsychiatric lupus: New mechanistic insights and future treatment directions. Nat. Rev. Rheumatol..

[4] Du Y., Sanam S., Kate K., Mohan C. (2015). Animal models of lupus and lupus nephritis. Curr. Pharm. Des..

[5] Ballok D.A. (2007). Neuroimmunopathology in a murine model of neuropsychiatric lupus. Brain Res. Rev..

[6] Desplat-Jégo S., Varriale S., Creidy R., Terra R., Bernard D., Khrestchatisky M., Izui S., Chicheportiche Y., Boucraut J. (2002). TWEAK is expressed by glial cells, induces astrocyte proliferation and increases EAE severity. J. Neuroimmunol..

[7] Stock A.D., Wen J., Putterman C. (2013). Neuropsychiatric lupus, the blood brain barrier, and the TWEAK/Fn14 pathway. Front. Immunol..

[8] Wen J., Xia Y., Stock A., Michaelson J.S., Burkly L.C., Gulinello M., Putterman C. (2013). Neuropsychiatric disease in murine lupus is dependent on the TWEAK/Fn14 pathway. J. Autoimmun..

[9] Fragoso-Loyo H., Atisha-Fregoso Y., Nuñez-Alvarez C.A., Llorente L. (2015). Utility of TWEAK to assess neuropsychiatric disease activity in systemic lupus erhytematosus. Lupus.

[10] Iacobas D.A., Iacobas S., Stout R., Spray D.C. (2020). Cellular environment remodels the genomic fabrics of functional pathways in astrocytes. Genes.

[11] Iacobas D.A. (2020). Biomarkers, master regulators and genomic fabric remodeling in a case of papillary thyroid carcinoma. Genes.

[12] Testing the Significance of Pearson’s r. https://webstat.une.edu.au/unit_materials/c6_common_statistical_tests/test_signif_pearson.html.

[13] Iacobas S., Ede N., Iacobas D.A. (2019). The Gene Master Regulators (GMR) approach provides legitimate targets for personalized, time-sensitive cancer gene therapy. Genes.

[14] Iacobas D.A., Iacobas S., Lee P.R., Cohen J.E., Fields R.D. (2019). Coordinated activity of transcriptional networks responding to the pattern of action potential firing in neurons. Genes.

[15] Arriens C., Wren J.D., Munroe M.E., Mohan C. (2016). Systemic lupus erythematosus biomarkers: The challenging quest. Rheumatology.

[16] Makinde H.M., Winter D.R., Procissi D., Mike E.V., Stock A.D., Kando M.J., Gadhvi G.T., Droho S., Bloomfield C.L., Dominguez S.T. (2020). A Novel microglia-specific transcriptional signature correlates with behavioral deficits in neuropsychiatric lupus. Front. Immunol..

[17] Iacobas D.A., Tuli N., Iacobas S., Rasamny J.K., Moscatello A., Geliebter J., Tiwari R.M. (2018). Gene master regulators of papillary and anaplastic thyroid cancer phenotypes. Oncotarget.

[18] Mathew R., Huang J., Iacobas S., Iacobas D.A. (2020). Pulmonary hypertension remodels the genomic fabrics of major functional pathways. Genes.

[19] Iacobas S., Iacobas D.A. (2010). Astrocyte proximity modulates the myelination gene fabric of oligodendrocytes. Neuron Glia Biol..

[20] Kanehisa M., Furumichi M., Tanabe M., Sato Y., Morishima K. (2017). KEGG: New perspectives on genomes, pathways, diseases and drugs. Nucleic Acids Res..

[21] KEGG Chemokine Signaling Pathway. https://www.kegg.jp/kegg-bin/show_pathway?mmu04062.

[22] KEGG PI3K-Akt Signaling Pathway. https://www.genome.jp/kegg-bin/show_pathway?mmu04151.

[23] KEGG Derived Glutamatergic Synapse. https://www.kegg.jp/kegg-bin/show_pathway?mmu04724.

[24] KEGG Derived GABAergic Synapse. https://www.kegg.jp/kegg-bin/show_pathway?hsa04727.

[25] KEGG Derived Cholinergic Synapse. https://www.kegg.jp/kegg-bin/show_pathway?hsa04725.

[26] KEGG Derived Dopaminergic Synpase. https://www.kegg.jp/kegg-bin/show_pathway?hsa04728.

[27] KEGG Derived Serotonergic Synapse. https://www.kegg.jp/kegg-bin/show_pathway?hsa04726.

[28] Mackay M., Vo A., Tang C.C., Small M., Anderson E., Ploran E.J., Storbeck J., Bascetta B., Kang S., Aranow C. (2019). Metabolic and microstructural alterations in the SLE brain correlate with cognitive impairment. JCI Insight.

[29] Ahmed M.S., Kovoor A., Nordman S., Abu Seman N., Gu T., Efendic S., Brismar K., Östenson C.-G., Gu H.F. (2012). Increased expression of adenylyl cyclase 3 in pancreatic islets and central nervous system of diabetic Goto-Kakizaki rats. Islets.

[30] Magro-Checa C., Steup-Beekman G.M., Huizinga T.W., Van Buchem M.A., Ronen I. (2018). Laboratory and neuroimaging biomarkers in neuropsychiatric systemic lupus erythematosus: Where do we stand, where to go?. Front. Med..

[31] Davenport E.E., Amariuta T., Gutierrez-Arcelus M., Slowikowski K., Westra H.-J., Luo Y., Shen C., Rao D.A., Zhang Y., Pearson S. (2018). Discovering in vivo cytokine-eQTL interactions from a lupus clinical trial. Genome Biol..

[32] Fortin S.P., Ennis M.J., Savitch B.A., Carpentieri D., McDonough W.S., Winkles J.A., Loftus J.C., Kingsley C., Hostetter G., Tran N.L. (2009). Tumor necrosis factor–like weak inducer of apoptosis stimulation of glioma cell survival is dependent on Akt2 function. Mol. Cancer Res..

[33] Iacobas D.A., Iacobas S., Spray D.C. (2007). Connexin-dependent transcellular transcriptomic networks in mouse brain. Prog. Biophys. Mol. Biol..

[34] Fan C., Iacobas D.A., Zhou D., Chen Q., Gavrialov O., Haddad G.G. (2005). Gene expression and phenotypic characterization of mouse heart after chronic constant and intermittent hypoxia. Physiol. Genom..

[35] Iacobas D.A., Iacobas S., Urban-Maldonado M., Scemes E., Spray D.C. (2008). Similar transcriptomic alterations in Cx43 knockdown and knockout astrocytes. Cell Commun. Adhes..

[36] Iacobas D.A., Iacobas S., Werner P., Scemes E., Spray D.C. (2007). Alteration of transcriptomic networks in adoptive-transfer experimental autoimmune encephalomyelitis. Front. Integr. Neurosci..

[37] Doerner J.L., Wen J., Xia Y., Paz K.B., Schairer D., Wu L., Chalmers S.A., Izmirly P., Michaelson J.S., Burkly L.C. (2015). TWEAK/Fn14 signaling involvement in the pathogenesis of cutaneous disease in the MRL/lpr model of spontaneous lupus. J. Investig. Dermatol..

[38] Michaelson J.S., Wisniacki N., Burkly L.C., Putterman C. (2012). Role of TWEAK in lupus nephritis: A bench-to-bedside review. J. Autoimmun..

[39] Hanahan D., Weinberg R.A. (2011). Hallmarks of cancer: The next generation. Cell.

[40] Engelman J.A. (2009). Targeting PI3K signalling in cancer: Opportunities, challenges and limitations. Nat. Rev. Cancer.

[41] Yang B., Yan P., Gong H., Zuo L., Shi Y., Guo J., Guo R., Xie J., Li B. (2016). TWEAK protects cardiomyocyte against apoptosis in a PI3K/AKT pathway dependent manner. Am. J. Transl. Res..

[42] Dogra C., Changotra H., Wedhas N., Qin X., Wergedal J.E., Kumar A. (2007). TNF-related weak inducer of apoptosis (TWEAK) is a potent skeletal muscle-wasting cytokine. FASEB J..

[43] Zhang F., Xu M., Yin X., Guo H., Zhang B., Wang Y., Xiao J., Zou X., Zhang M., Zhuge Y. (2019). TWEAK promotes hepatic stellate cell migration through activating EGFR/Src and PI3K/AKT pathways. Cell Biol. Int..

[44] Xu R.-D., Feng F., Yu X.-S., Liu Z.-D., Lao L.-F. (2018). miR-149-5p inhibits cell growth by regulating TWEAK/Fn14/PI3K/AKT pathway and predicts favorable survival in human osteosarcoma. Int. J. Immunopathol. Pharmacol..

[45] Tinahones F.J., C-Soriguer F.J., Mancha I., Esteva I. (1996). Malignant paraganglioma with aortic infiltration cured by radical surgery. Ann. Med. Interna.

[46] Bishop G.A., Berbari N.F., Lewis J., Mykytyn K. (2007). Type III adenylyl cyclase localizes to primary cilia throughout the adult mouse brain. J. Comp. Neurol..

[47] Chen X., Luo J., Leng Y., Yang Y., Zweifel L.S., Palmiter R.D., Storm D.R. (2016). Ablation of type III adenylyl cyclase in mice causes reduced neuronal activity, altered sleep pattern, and depression-like phenotypes. Biol. Psychiatry.

[48] Speliotes E.K., Willer C.J., Berndt S.I., Monda K.L., Thorleifsson G., Jackson A.U., Allen H.L., Lindgren C.M., Luan J., Mägi R. (2010). Association analyses of 249,796 individuals reveal 18 new loci associated with body mass index. Nat. Genet..

[49] Warrington N., Howe L.D., Paternoster L., Kaakinen M., Herrala S., Huikari V., Wu Y.Y., Kemp J.P., Timpson N.J., Pourcain B.S. (2015). A genome-wide association study of body mass index across early life and childhood. Int. J. Epidemiol..

[50] Grarup N., Moltke I., Andersen M.K., Dalby M., Vitting-Seerup K., Kern T., Mahendran Y., Jørsboe E., Larsen C.V.L., Dahl-Petersen I.K. (2018). Loss-of-function variants in ADCY3 increase risk of obesity and type 2 diabetes. Nat. Genet..

[51] Gonzales M.M., Tarumi T., Miles S.C., Tanaka H., Shah F., Haley A.P. (2010). Insulin sensitivity as a mediator of the relationship between BMI and Working memory-related brain activation. Obesity.

[52] Su F., Shu H., Ye Q., Wang Z., Xie C., Yuan B., Zhang Z., Bai F. (2017). Brain insulin resistance deteriorates cognition by altering the topological features of brain networks. Neuroimage Clin..

[53] Zou J., Wu K., Lin C., Jie Z.-G. (2020). LINC00319 acts as a microRNA-335–5p sponge to accelerate tumor growth and metastasis in gastric cancer by upregulating ADCY3. Am. J. Physiol. Liver Physiol..

[54] Pullabhatla V., Roberts A.L., Lewis M.J., Mauro D., Morris D.L., Odhams C.A., Tombleson P., Liljedahl U., Vyse S., Simpson M.A. (2018). De novo mutations implicate novel genes in systemic lupus erythematosus. Hum. Mol. Genet..

[55] Pakzad B., Shirpour R., Mousavi M., Karimzadeh H., Salehi A., Kazemi M., Amini G., Akbari M., Salehi R. (2020). C1QTNF4 gene p.His198Gln mutation is correlated with early-onset systemic lupus erythematosus in Iranian patients. Int. J. Rheum. Dis..

[56] Iacobas D.A., Iacobas S., Thomas N., Spray D.C. (2010). Sex-dependent gene regulatory networks of the heart rhythm. Funct. Integr. Genom..

[57] Thomas N.M., Jasmin J.F., Lisanti M.P., Iacobas D.A. (2011). Sex differences in expression and subcellular localization of heart rhythm determinant proteins. Biochem. Biophys. Res. Commun..

